# Distúrbio mineral e ósseo: prevalencia subestimada nos estágios iniciais da doenga renal crónica

**DOI:** 10.15649/cuidarte.2266

**Published:** 2023-03-29

**Authors:** Karla Amaral Nogueira-Quadros, Flávio Augusto-de-Morais, Francisco Edson Coelho-de-Vasconcelos, Yoshimi José Ávila-Watanabe, Allan de Morais-Bessa, Fernanda Marcelino de-Rezende-e-Silva, Joao Victor Marques-Guedes, Vinícius Silva-Belo, Clareci Silva-Cardoso, Alba Otoni

**Affiliations:** 1 . Universidade Federal de Sao Joao Del Rei (UFSJ) campus Divinópolis/MG, Divinópolis/MG/Brasil. Email: karla.quadros@uemg.br Universidade Federal de São João del-Rei Universidade Federal de Sao Joao Del Rei Brazil karla.quadros@uemg.br; 2 . Secretaria Municipal de Saúde de Divinópolis/MG, Divinópolis/MG/Brasil. Email: drfamorais@gmail.com Secretaria Municipal de Saúde de Divinópolis Secretaria Municipal de Saúde de Divinópolis Brazil drfamorais@gmail.com; 3 . Complexo Hospitalar Sao Joáo de Deus de Divinópolis/MG, Divinópolis/MG/Brasil. Email: fecvasconcelos@uol.com.br Complexo Hospitalar Sao Joáo de Deus de Divinópolis Brasil fecvasconcelos@uol.com.br; 4 . Universidade Federal de Sao Joáo Del Rei (UFSJ) campus Divinópolis/MG, Divinópolis/MG/Brasil. Email: yosh.watanabe@gmail.com Universidade Federal de São João del-Rei Universidade Federal de Sao Joáo Del Rei Brazil yosh.watanabe@gmail.com; 5 . Universidade do Estado de Minas Gerais (UEMG) unidade de Divinópolis/MG, Divinópolis/MG/Brasil. Email: allanmoraisone@hotmail.com Universidade do Estado de Minas Gerais Universidade do Estado de Minas Gerais Brazil allanmoraisone@hotmail.com; 6 . Universidade Federal de Sao Joáo Del Rei (UFSJ) campus Divinópolis/MG, Divinópolis/MG/Brasil. Email: fernanda.silva@gmail.com Universidade Federal de São João del-Rei Universidade Federal de Sao Joáo Del Rei Brazil; 7 . Universidade Federal de Sao Joao Del Rei (UFSJ) campus Divinópolis/MG, Divinópolis/MG/Brasil. Email: jvmg92@gmail.com Universidade Federal de São João del-Rei Universidade Federal de Sao Joao Del Rei Brazil jvmg92@gmail.com; 8 . Universidade Federal de Sao Joao Del Rei (UFSJ) campus Divinópolis/MG, Divinópolis/MG/Brasil. Email: viniciusbelo4@hotmail.com Universidade Federal de São João del-Rei Universidade Federal de Sao Joao Del Rei Brazil viniciusbelo4@hotmail.com; 9 . Universidade Federal de Sao Joao Del Rei (UFSJ) campus Divinópolis/MG, Divinópolis/MG/Brasil. Email: clarecicardoso@yahoo.com.br Universidade Federal de São João del-Rei Universidade Federal de Sao Joao Del Rei Brazil clarecicardoso@yahoo.com.br; 10 . Universidade Federal de Sao Joao Del Rei (UFSJ) campus Divinópolis/MG, Divinópolis/MG/Brasil. Email: albaotoni@ufsj.edu.br Universidade Federal de São João del-Rei Universidade Federal de Sao Joao Del Rei Brazil albaotoni@ufsj.edu.br

**Keywords:** Distúrbio Mineral e Ósseo na Doenca Renal Crónica, Doenca renal crónica, Falencia Renal Crónica, Insuficiencia Renal Crónica, Hiperparatireoidismo Secundário., Chronic Kidney Disease-Mineral and Bone Disorder, Renal Insufficiency, Chronic, Kidney Failure, Chronic, Hyperparathyroidism, Secondary., Trastorno Mineral y Óseo Asociado a la Enfermedad Renal Crónica, Insuficiencia Renal Crónica, Fallo Renal Crónico, Hiperparatiroidismo secundario.

## Abstract

**Introdujo:**

o objetivo foi avaliar a prevalencia do distúrbio mineral e ósseo em pacientes com doenca renal crónica e a associate entre Taxa de Filtrado Glomerular estimada (TFGe) e os indicadores do distúrbio mineral e ósseo (DMO) (cálcio, fósforo e PTH) em pacientes renais crónicos nao dialíticos.

**Materiais e Métodos:**

estudo seccional da linha de base de uma coorte de dois anos, com adultos e idosos renais crónicos em tratamento conservador. Para identificado do DMO utilizamos os seguintes valores séricos: PTH (> 150 pg/mL) e/ou hipocalcemia (Ca < 8,8mg/dl) e/ou hiperfosfatemia (P > 4,6 mg/dl). Na análise estatística utilizou-se: regressao de Poisson; T de Student, Mann Whitney e correlacóes de Pearson e Spearman. Nivel de significancia foi de 5%.

**Resultados:**

prevalencia de DMO de 54,6% (n=41) (IC 95%: 43,45 - 65,43). A maior prevalencia de DMO foi em pessoas do sexo feminino, alfabetizadas, idosas, nao etilistas, nao tabagistas, sedentárias e de cor de pele branca, porém, sem diferenca estatística entre os grupos com e sem DMO. As correlacóes entre P e PTH com TFGe foram significativas, inversas, de forca moderada (p= <0,005 e p = 0,003; coeficientes de correlato = - 0,312 e - 0,379 respectivamente).

**Discussao:**

os achados desse estudo mostraram que existe uma lacuna no acompanhamento do DMO-DRC pela atencao primária e a prática clínica deve ser revista.

**Conclusao:**

identificou-se prevalencia robusta de DMO nos estágios precoces da DRC, além de correlacóes significativas entre o aumento dos níveis de fósforo e PTH e piora das funcóes renais.

## Abstract (en)

**Introduction:**

mineral and bone disorder (BMD) is a serious complication of chronic kidney disease (CKD) that increases risks for death from cardiovascular causes and impairs quality of life of affected patients.

**Objective:**

to evaluate the prevalence of BMD in patients with CKD and the association between estimated Glomerular Filtration Rate (eGFR) and BMD indicators (calcium, phosphorus and PTH) in non-dialysis patients.

**Materials and Methods:**

sectional study of a two-year cohort of chronic renal adults and elderly patients on conservative treatment. BMD was identified by serum values of: PTH (> 150 pg/mL) and/or hypocalcemia (Ca < 8.8mg/dl) and/or hyperphosphatemia (P > 4.6 mg/dl). The statistical analysis used: Poisson regression; Student's T, Mann Whitney and Pearson and Spearman correlations with 5% significance level.

**Results:**

BMD prevalence was 54.6% (n=41) (95% CI: 43.45 - 65.43), more frequent in women, literate, elderly, non-drinkers, non-smokers, sedentary and white skin color. Correlations between P and PTH with GFRe were significant, inverse, moderate strength (p= <0.005 and p = 0.003; correlation coefficients = - 0.312 and - 0.379 respectively). the findings of this study highlighted gaps in the monitoring of BMD-DRC by primary care, requiring a review of clinical practices.

**Conclusion:**

robust prevalence of BMD in the early stages of CKD was identified, in addition to correlations between increased phosphorus and PTH levels and worsening kidney function.

## Resumen

**Introducción:**

el trastorno mineral y óseo (TMO) es una complicación grave de la enfermedad renal crónica (ERC) que aumenta el riesgo de muerte por causas cardiovasculares y deteriora la calidad de vida de los pacientes afectados.

**Objetivo:**

evaluar la prevalencia de la DMO en pacientes con RDC y la asociación entre la tasa de filtración glomerular estimada (TFGe) y los indicadores de DMO (calcio, fósforo y PTH) en pacientes no dialíticos.

**Materiales y métodos:**

estudio seccional de una cohorte de dos años de pacientes renales crónicos adultos y ancianos en tratamiento conservador. La DMO se identificó por los valores séricos de: PTH (> 150 pg/mL) y/o hipocalcemia (Ca < 8,8mg/ dl) y/o hiperfosfatemia (P > 4,6 mg/dl). El análisis estadístico utilizado: regresión de Poisson; T de Student, Mann Whitney y correlaciones de Pearson y Spearman con un nivel de significación del 5%.

**Resultados:**

la prevalencia de DMO fue del 54,6% (n=41) (IC 95%: 43,45 - 65,43), más frecuente en mujeres, alfabetizadas, de edad avanzada, no bebedoras, no fumadoras, sedentarias y de color de piel blanca. Las correlaciones entre el P y la PTH con el GFRe fueron significativas, inversas, de fuerza moderada (p= <0,005 y p = 0,003; coeficientes de correlación = - 0,312 y - 0,379 respectivamente).

**Discusión:**

los resultados de este estudio evidencian lagunas en el seguimiento de la DMO-DRC por parte de la atención primaria, lo que requiere una revisión de las prácticas clínicas. **Conclusión:** se identificó una fuerte prevalencia de la DMO en las primeras fases de la ERC, además de correlaciones entre el aumento de los niveles de fósforo y PTH y el empeoramiento de la función renal.

## Introdujo

A doenga renal crónica (DRC) é um importante problema de saúde pública em escala mundial, com taxa de prevaléncia crescente, afetando 10% da populagao adulta em todo o mundo e mais de um tergo dos idosos^1^. Em estudo recente desenvolvido com populagao adulta brasileira em 2019, identificou-se prevaléncia de 6,7% de Taxa de Filtragao Glomerular estimada (TFGe) alterada^2^. E em Coorte também brasileira envolvendo 15.000 funcionários de seis instituigóes públicas de ensino superior, a prevaléncia da TFGe alterada foi ainda maior, de 9,9%^3^.

É consenso na literatura que quanto mais precoce a descoberta do comprometimento renal, mais rápidas também poderao ser as intervengóes preventivas no sentido de amenizar a evolugao das lesóes e minimizar as complicagóes inerentes ao quadro evolutivo para a doenga renal terminal^4^^,^^5^^,^^6^.

Entre as complicagóes associadas a instalagao da DRC está o Distúrbio Mineral e Ósseo (DMO). Á medida que ocorre a perda da Taxa de Filtragao Glomerular (TFG), o metabolismo mineral torna-se perturbado piorando a microestrutura e a remodelagao óssea. Essa associagao simultanea de perda das fungóes renais e perturbagóes no metabolismo mineral e ósseo é conhecida como DMO-DRC, e tem, de uma maneira geral, como fatores de risco o sexo feminino, idade avangada, menopausa precoce, histórico de osteoporose ou de fratura osteoporótica no indivíduo e progenitores, baixa ingesta de cálcio e vitamina D3 e hábitos de vida nao saudáveis^6^.

Uma vez iniciado, o DMO-DRC caracteriza-se pela evolugao de distúrbios clínicos e bioquímicos com alteragóes nos níveis de Fator de Crescimento de Fibroblastos 23 (FGF23) /klotho, cálcio, fosfato, hormónio paratireóideo e vitamina D ativa, além das anormalidades ósseas (remodelagao óssea, mineralizagao e volume ósseo). Como valores laboratoriais de referéncia para classificagao DMO- DRC cita-se PTH > 150 pg/mL e/ou Cálcio (Ca) < 8,8mg/dl e/ou fósforo (p) > 4,6 mg/dl). Tais alteragóes evolutivas podem culminar em aumento dos riscos para fraturas, desenvolvimento de doengas cardiovasculares e morte^6^^,^^7^.

Apesar das evidéncias apontarem para o desenvolvimento do DMO ainda nos estágios precoces da DRC com avango de consequente comprometimento cardiovascular devastador em pacientes já em terapias renais substitutivas, os estudos com esse foco em pacientes pré-dialíticos pouco avangaram em todo mundo. Em interessante trabalho americano (2021), os autores alertaram sobre o difícil e restrito diagnóstico diferencial dos distúrbios minerais e ósseos associados ao rim. Segundo eles nem exames laboratoriais, nem as investigagóes diagnósticas nao invasivas podem discriminar a osteoporose das várias formas da osteodistrofia renal. E registram que terapeuticamente, nenhum tratamento para fraturas, consequentes ao DMO-DRC foi aprovado pelo Food and Drug Administration (FDA).

Sugerem que diante de um cenário ainda intrincado do DMO-DRC, as abordagens clássicas a partir do máximo conhecimento sobre o mecanismo fisiopatológico desse distúrbio conforme o estágio da DRC, bem como opgóes off label em determinadas situagóes, como por exemplo, para tratar os estágios 3b-5 da DRC em pacientes de alto risco, podem ser a melhor diregao para uma abordagem terapéutica mais assertiva^8^. Em outro estudo indiano recente, os autores confirmaram as alteragóes ósseas de alto turnover como frequentes nos estágios pré-dialíticos, bem como fatores de risco para alteragóes ósseas (hiperparatireoidismo, a hiperfosfatemia e a deficiéncia de vitamina D). E em pacientes com DRC em diálise, encontraram alta prevaléncia da doenga óssea adinamica.

Estes autores sugerem que as alteragóes ósseas parecem ser advindas de uma dificuldade de abordagem terapéutica eficiente e um consequente uso imprudente de aglutinantes de fosfato (especialmente contendo cálcio) e análogos da vitamina D^9^. E assim como os autores americanos recomendam uma abordagem cuidadosa para diagnosticar, prevenir e tratar as várias anormalidades minerais ósseas em pacientes com DRC. Recentemente no Brasil com o intuito de acompanhar as tendencias mundiais de abordagens terapéuticas ao DMO-DRC foi publicada uma atualizagao das diretrizes brasileiras para o tratamento e avaliagao do distúrbio mineral e ósseo da doenga renal crónica.^6^ Mesmo assim, ainda é notório que o cenário das condigóes ósseas associadas ao rim em pessoas brasileiras com DRC em estágios nao dialíticos ainda permanece latente e requer estudos clínicos pautados em rigor metodológico para o reconhecimento do contexto peculiar da realidade _nacional_^10^^,^^11^^,^^12^_._

Neste sentido, considerando que o reconhecimento das particularidades da populagao brasileira com DMO-DRC possa contribuir para efetivagao de políticas públicas na prática clínica assistencial em todos os níveis de atengao a saúde, em especial, com foco na abordagem preventiva na atengao primária a saúde ( APS) e ainda visando redugao do custo dos recursos públicos destinados a tratar os efeitos das complicagóes advindas da DMO-DRC, o objetivo desse estudo foi avaliar a prevaléncia de DMO-DRC, bem como a associagao entre TFGe e os indicadores de DMO (cálcio, fósforo e PTH) em pacientes renais crónicos nao dialíticos.

## Materiais e Métodos

### Delineamento e Populagáo do estudo

Trata-se de um estudo quantitativo, transversal e analítico sendo um recorte temporal de uma coorte. Para delineamento do estudo utilizou-se as recomendagóes do Strengthening the Reporting of Observational Studies in Epidemiology Statement (STROBE^13^^,^^14^.

A populagao elegível foi composta por pacientes que compareceram para atendimento no ambulatório de nefrologia no período entre junho de 2018 a junho de 2019. Como critérios de inclusao foram considerados: pacientes adultos e idosos, de ambos os sexos, que tiveram o diagnóstico de DRC confirmado ainda em estágios nao dialíticos e que tiveram informagóes no prontuário compatíveis com a identificagao do distúrbio mineral e ósseo (cálcio, fósforo e Paratormónio (PTH).

### Coleta de dados e Variáveis de interesse do estudo

A coleta de dados ocorreu no ambulatório de nefrologia no período de junho de 2018 a junho de 2019. As informagóes validadas foram exportadas para o pacote estatístico Stata/MP versao 17.0 para processamento de dados. O banco de dados foi armazenado no Harvard Dataverse, V1^15^. Foram realizadas entrevistas guiadas por protocolo de pesquisa elaborado pelos pesquisadores tendo o próprio paciente como informante. Ao comparecer para admissao e posterior acompanhamento no ambulatório municipal de nefrologia, que ocorria duas vezes por semana, todos os pacientes elegíveis foram convidados a participar da pesquisa. Uma ante sala do consultório do nefrologista foi preparada com mesa, cadeira e privacidade para a abordagem a esses pacientes. Todos aqueles que preencheram os critérios de inclusao, inclusive com apresentagao dos exames para caracterizagao do DMO e aceitaram participar foram incluídos na pesquisa. E para obtengao dos resultados dos exames laboratoriais, identificados na primeira consulta, ou complementagao de dados faltantes, os prontuários foram consultados como fonte secundária de informagao.

O DMO foi classificado quando o PTH > 150 pg/mL (quimiluminescéncia método - pg/mL) e/ou Cálcio (Ca) < 8,8mg/dl (método Arsenazo III - mg/dL) e/ou fósforo (p) > 4,6 mg/dl) (método de fosfomolibidato UV - mg/dL^6),(^^7^.

As outras variáveis foram organizadas em blocos: 1) sociodemográficas: sexo, idade, estado civil, escolaridade e cor da pele; 2) comportamento em saúde: etilismo, tabagismo e sedentarismo^16^^,^^17^^,^^18^; 3) clínicas: registro da doenga de base da DRC, comorbidades, Índice de Massa Corporal (IMC)^18^, Pressáo Arterial Sistólica (PAS), Pressao Arterial Diastólica (PAD)^19^^,^^20^histórico familiar para doenga nefrológica, estágio atual da DRC e uso de medicamentos de forma categorizada como total de medicamentos até cinco e acima de cinco independente da classe medicamentosa.

Os valores da TFGe utilizados para diagnóstico e classificagáo do estágio da DRC foram retirados do prontuário na data do diagnóstico de DRC. Como referéncia para o cálculo da TFGe os profissionais do ambulatório de nefrologia utilizaram a aplicagáo da fórmula Chronic Kidney Disease Epidemiology Collaboration (CKD-EPI) para maiores de 18 anos, que considera as variáveis creatinina sérica, raga ou cor negra, idade e sexo^20^. Salienta-se que em estudo publicado em 2016 no Brasil, os dados relacionados a raga para cálculo da TFGe náo acrescentam informagóes adequadas para a populagáo brasileira, visto a grande diversidade e miscigenagáo genotípica da populagáo, e por isso, essa variável náo é utilizada na rotina de assisténcia aos pacientes renais no ambulatório, náo sendo, portanto, utilizada para o referido cálculo nesse estudo^21^. Classificamos como TFGe reduzida quando os valores calculados foram < 60 mL/ min/1,73m2.^16^

### Análise estatística

Para caracterizagáo do perfil sociodemográfico, clínico e laboratorial da populagáo foi utilizada estatística descritiva por meio de medidas de frequéncia e dispersáo. Calculou-se a razáo de prevaléncia para cada variável e o seu respectivo intervalo de confianga pelo modelo de Poisson com variancias robustas, uma vez que a frequéncia do desfecho foi > 30%. No modelo de Poisson bivariado náo houve associagáo com p < 0,20 e, por isso, náo foi realizada regressáo de Poisson multivariada. Os testes T de Student e Mann-Whitney foram utilizados para identificar as diferengas das médias/medianas dos níveis séricos de cálcio, fósforo e PTH nos estágios de DRC, bem como para verificar existéncia de diferengas das médias de TFGe entre os pacientes com e sem DMO, conforme a distribuigáo de normalidade (teste de Shapiro-Wilk). As correlagóes de Pearson e Spearman (teste de Shapiro-Wilk) foram utilizadas para avaliar a correlagáo entre TFGe e os indicadores de DMO (cálcio, fósforo e PTH). Para avaliar a associagáo entre as variáveis explicativas qualitativas e a presenga ou náo do DMO foi utilizado Qui-quadrado de Pearson.

## Resultados

Dos 262 pacientes atendidos na Policlínica no período de um ano de coleta, 75 atenderam os critérios de elegibilidade e foram incluídos no estudo. A auséncia de exames para o diagnóstico de DMO (cálcio e/ou fósforo e/ou PTH) foi o motivo da exclusáo de 187 pacientes. A caracterizagáo do perfil sociodemográfico, clínico e laboratorial da populagáo encontra-se descrita na Tabela 1.

**Tabela 1:** Variáveis sociodemográficas e estilo de vida dos pacientes com DMO-DRC do ambulatório de nefrologia de um município do centro oeste mineiro/Brasil, no período entre junho/2018 a junho/2019 (n= 75).

	DMO 83. 84.		
	Sem DMO (n=34)	Com DMO (n=41)	RP* (IC**95%)
	n (%)	n (%)	
Sexo			
Feminino	14(36,8%)	24(63,1%)	1,0
Masculino	20(54,0%)	17(45,9%)	1,47 [0,88-2,45]
Idade			
Menos de 60 anos	4(40,0%)	6(60,0%)	1,0
60 anos ou mais	30(46,1%)	35(53,8%)	0,89 [0,81-1,56]
Estado civil			
Sem companheiro	20(50,0%)	20(50,0%)	1,0
Com companheiro	14(40,0%)	21(60%)	0,86 [0,55 -1,26]
Escolaridade			
Alfabetizado	28(45,9%)	33(54,0%)	1,0
Nao alfabetizado	6(42,8%)	8(57,1%)	1,05 [0,63- 1,76]
Cor da pele			
Branca	25(47,1%)	28(52,8%)	1,0
Preta	2(22,2%)	7(77,7%)	1,47 [0,55 -2,27]
Parda	7(53,8%)	6(46,1%)	0,87 [0,45 -1,66]
Etilismo			
Nao	30(38,4%)	38(48,7%)	1,0
Sim	4(57,1%)	3(42,8%)	0,76 [0,31 -1,86]
Tabagismo			
Nao	32(45,0%)	39(54,9%)	1,0
Sim	2(50,0%)	2(50,0%)	0,91 [0,33 -2,49]
Sedentarismo			
Nao	9(36,0%)	16(64,0%)	1,0
Sim	25(50,0%)	25(50,0%)	0,78 [0,53-1,17]

* Razao de Prevalencia/Regressao de Poisson; **IC: intervalo de confianza; DMO- DRC: distúrbio Mineral e ósseo associado a DRC. Fonte: Dados da Pesquisa, 2018-2019.

*A* prevaléncia de DMO na populagáo total do estudo foi de 54,6% (41/75) (IC 95%: 43,45 - 65,43). No que diz respeito a doengas de base da DRC, foi identificada a doenga renal do diabetes em 44 (58,6%) dos pacientes e, embora náo confirmado em prontuário o diagnóstico de nefropatia hipertensiva, em 66 (88%) pacientes havia registro de diagnóstico de hipertensáo arterial sistémica (HAS) prévio ao diagnóstico de DRC. Como comorbidades mais frequentes foi identificado: história de insuficiéncia cardíaca congestiva em 19 pacientes (25,3%), hipotireoidismo em 12 (16,0%), depressáo em nove (12%), além de outras menos frequentes.

Ao comparar os níveis de cálcio, fósforo e PTH entre os estágios 3a; 3b e 4 da DRC, apenas a média do fósforo mostrou diferenga estatística (p=0,02) sendo a maior média encontrada no estágio 4 (média de 4,1 mg/dL± 0,77), porém, sem ultrapassar níveis considerados fisiológicos. A média de PTH também foi maior no estágio 4 (116,5 pg/mL) e o cálcio manteve-se em 9,4 mg/dL nos tres estágios. (Tabela 2).

**Tabela 2:** Comparagáo entre as médias dos níveis de cálcio, fósforo e PTH entre os estágios 3a; 3b e 4 da DRC dos pacientes com Distúrbio Mineral e ósseo - doenga renal crónica do ambulatório de nefrologia de um município do centro oeste mineiro/Brasil, junho de 2018 a junho de 2019 (n=75).

Marcadores de DMO	Estágios da doenga renal		P valor*
	3a e 3b	4	
Fósforo(mg/dL)	3,6	4,1	0,02*
Cálcio (mg/dL)	9,4	9,4	0,58
PTH (pg/mL)	109,3	116,5	0,07

* Teste de t de Student

Identificamos ainda hiperfosfatemia (P > 4,6 mg/dl) em 14 pacientes (18,6%); níveis de PTH > 150pg/ ml em oito pacientes (10,7%) e hipocalcemia (< 8,8mg/dl) em 19 pacientes (25,3%).

As variáveis clínicas e laboratoriais nao apresentaram associagao significativa com o DMO. Salienta- se, no entanto, que a maior frequencia de DMO foi identificada em pacientes com estágios mais avangados da DRC (3b e 4), bem como em pacientes com o maior consumo de medicamentos (acima de 5 medicamentos). Com relagao a média de TFGe esta foi discretamente maior (32, 5ml/min/1,73 m2) no grupo sem DMO quando comparado com o grupo com DMO, porém, também sem diferenga estatística.

Quando buscamos identificar a correlagao entre a TFGe quantitativa com cada um dos marcadores de DMO (cálcio, o fósforo e o PTH), identificamos que: as correlagóes entre P e PTH com TFGe foram significativas, inversas, de forga moderada, sendo coeficientes de correlagao igual a - 0,312 e - 0,379 respectivamente, ou seja, o fósforo e o PTH séricos aumentavam a medida que a TFGe diminuía. A correlagao entre cálcio e TFGe nao foi significativa (Tabela 3).

**Tabela 3:** Correlagáo entre TFGe e cálcio, fósforo e paratormónio dos pacientes com Distúrbio Mineral e ósseo - doenga renal crónica do ambulatório de nefrologia de um municipio do centro oeste mineiro/Brasil, junho de 2018 a junho de 2 (n=75).

Marcadores de distúrbio mineral e ósseo	Coeficiente de Correlagao	Valor-p***
Fósforo*	- 0,312	<0,001
Cálcio **	0,033	0,393
Paratormónio*	- 0,379	0,003

*Correlagáo de Pearson**Correla^ao de Spearman***Significancia estatística p<0,05

## Discussao

Identificamos uma prevaléncia de 54,6% de DMO em pacientes renais crónicos nao dialíticos, sendo os estágios 3b e 4 da DRC os mais acometidos, porém, sem diferenga estatística entre eles. A literatura referente a presenga do DMO em pacientes com DRC em estágio terminal em TRS, é ampla^12^^,^^22^^-^^24^. No entanto, nao encontramos estudos nacionais que avaliaram o DMO nos estágios mais precoces da DRC no Brasil. Destaca-se que mesmo frente ao índice de mais de 50% de DMO na populagao estudada, nenhum dos pacientes até aquele momento de atendimento pelo nefrologista, tinha conhecimento dessa condigao de saúde e nao encontramos nos prontuários qualquer registro relativo a essas alteragóes.

O modelo assistencial para os pacientes do nosso estudo antes de chegarem para a avaliagao da equipe de nefrologia, preconiza uma assisténcia prévia na atengao primária de saúde^6^. Segundo as diretrizes do Ministério da Saúde do Brasil (2014), reafirmados pela atualizagao das diretrizes DMO- DRC (2021), por meio dessa assisténcia, os pacientes deveriam ser abordados com uma terapéutica que implicasse no acompanhamento das alteragóes do metabolismo mineral e ósseo já a partir do estágio 3a^6^^,^^16^. A dosagem anual do fósforo e do PTH intacto é recomendada e, se identificado alteragóes, mesmo ainda na atengao primária, devem ser discutidas com o nefrologista referéncia da Unidade Básica de Saúde.

Diante do desconhecimento por parte dos pacientes e da falta de registro dos elementos primordiais na identificagao do DMO nos prontuários, os autores desse estudo refletem se o acompanhamento na atengao primária no município estudado ocorre de forma ampla a abordar todas as possíveis complicagóes que se instalam simultaneas as alteragóes das fungóes renais. Do total de 262 pacientes com DRC em estágios 3a, 3b e 4 encaminhados pela atengao primária para atendimento pela primeira vez na nefrologia durante o período de coleta da investigagao, 187 (72,9%) nao puderam ser incluídos no estudo pela falta de registro de exames laboratoriais que permitissem a identificagao do DMO.

É de suma importancia que as equipes de saúde da atengao primária incorporem as suas atividades diárias de trabalho a busca ativa ao paciente com DRC ainda em estágio precoce de comprometimento renal e realizem um rigoroso acompanhamento para identificar, o mais breve possível, as complicagóes inerentes a perda evolutiva das fungóes renais. E mais, que invistam esforgos nas propostas de intervengao a partir da identificagao imediata da alteragao do metabolismo cálcio e fósforo. Entre as medidas preventivas que poderiam ser utilizadas com esses pacientes, cita-se: abordagens nao farmacológicas, como dieta controlada de fósforo e de cálcio, estímulo a hábitos saudáveis de vida até o uso de medicamentos quelantes de fósforo (carbonato de cálcio, acetato de cálcio e sevelamer), vitamina D ativa (calcitriol oral e venoso), análogos de vitamina D (alfacalcidol), ativadores seletivos do receptor de vitamina D (paricalcitol), e calcimiméticos (cinacalcete).

Em 2013 foram publicados resultados de uma coorte de quatro anos realizada a partir de dados de um ensaio clínico randomizado de Doengas Renais e Hipertensao Arterial^4^ com 809 afro americanos, e embora nao tenham identificado a prevaléncia do DMO, avaliaram a associagao dos níveis basais de metabólitos minerais: PTH, fósforo e o Fator de Crescimento de Fibroblastos 23 (FGF23) com risco de evolugao para DRC terminal ou morte. Os autores encontraram uma correlagao inversa entre a TFGe e a presenga de DMO, ou seja, participantes com declínios mais rápidos na TFG tiveram os maiores aumentos nos parametros séricos daqueles metabólitos minerais (P <0,01 para cada). Os nossos resultados corroboram esses achados e mostram que os indicadores fósforo e PTH também tiveram correlagao significativa, inversa de forga moderada com o declínio da fungao renal (FR). Tanto os resultados do estudo americano quanto do nosso estudo indicam que o DMO acontece efetivamente já nos estágios precoces da DRC, de forma mais evidente nos estágios 3b e 4, e, portanto, a abordagem preventiva é imprescindível.

Cabe ressaltar, no entanto, que se acredita que na realidade a identificado de níveis aumentados de fósforo e PTH como preditores do DMO, mesmo nos estágios nao dialíticos da DRC, é uma agáo tardia no que diz respeito ao início efetivo do DMO. Isso é dito porque, sabidamente o FGF23, a partir da ligagáo com o receptor FGFR, intermediada pelo correceptor proteína Klotho, tem como principal fundo fisiológica regular o metabolismo do fosfato sérico, os níveis séricos da 1,25-dihidroxivitamina D e a mineralizado óssea^22^^-^^25^. Assim, associado as alteragóes renais próprias da etiologia basal, entende-se que previamente os metabolismos alterados da síntese de FGF23 nos osteócitos (níveis séricos aumentados) e do Klotho nos túbulos renais distais (diminuídos nos níveis séricos) é que elevam os níveis séricos do fósforo e, portanto, antecedem a hiperfosfatemia. Sendo provavelmente, FGF23 o preditor mais precoce do DMO.

Considerando que o FGF23 é secretado e facilmente detectável no plasma de pessoas saudáveis^25^, talvez fosse o momento de se investir em pesquisas que pudessem confirmar as relagóes entre FGF23, PTH e fósforo para diagnóstico precoce do DMO-DRC. A utilizagáo dos níveis séricos de FGF23 na prática clínica poderia minimizar de forma muito precoce potenciais complicagóes da DRC, inclusive controlando seus desfechos mais graves como a instalagáo de doengas cardiovasculares, além da probabilidade de melhoria do prognóstico. Embora audacioso, náo seria infundada a ideia de repensar as diretrizes para abordagem aos pacientes em risco de desenvolver essa condigáo de saúde (DMO-DRC) e oferecer, para além de pesquisas, o acesso via sistema público de saúde, o exame dos níveis séricos do FGF23.

Retomando a interpretagáo dos resultados acessíveis, em marcante estudo Tentori et al (2008) já alertavam para o aumento da morbimortalidade na presenga de anormalidades nos níveis séricos de cálcio, fósforo e PTH, sendo maior o risco, quanto maior fossem os níveis desses metabólitos. Esses autores registraram que o agravamento dos distúrbios de cálcio, fósforo e PTH promoveram a instalagáo do hiperparatireoidismo secundário (HPT2) associado a DRC, e destacaram que quando náo controlada, essa associagáo DMO-DRC pode conduzir a maciga calcificagáo vascular, doenga cardiovascular e morte^26^. No nosso estudo, identificamos alteragóes tanto no fósforo, quanto no cálcio e no PTH ainda sem qualquer abordagem da equipe de saúde no sentido de controlar esses níveis para prevenir efeitos indesejáveis.

Tomando como base essas afirmagóes, embora parega evidente, enquanto o exame de FGF23 náo é consolidado e disponibilizado como marcador precoce de DMO-DRC, é importante ressaltar que os níveis séricos de Cálcio, fósforo, PTH além da vitamina D, devem ser acompanhados rigorosamente, conforme recomenda o MS do Brasil (2014^16)^ e o KDIGO 2017^7^, desde as fases náo dialíticas da DRC.

Por fim, os achados desse estudo mostraram que existe uma lacuna no acompanhamento do DMO- DRC pelas equipes da atengáo primária e, portanto, a prática clínica aplicada a esses pacientes deve ser revista a fim de alcangar a excelencia proposta pelo sistema único de saúde. Mesmo diante de resultados que contribuem para a assistencia direta ao paciente com DRC náo dialítica é preciso registrar as limitagóes do estudo: o delineamento seccional náo permite avaliar a relagáo causa e efeito devido aos vieses característicos de estudos transversais. Além disso, náo foi possível avaliar o FGF 23, em fungáo da náo disponibilidade desse exame pelo sistema público de saúde e falta de financiamento da pesquisa por fontes de fomento. Também náo foi possível inserir variáveis de interesse do estudo devido a náo disponibilizagáo desses dados nos prontuários e no sistema de informado do municipio. Por este mesmo motivo (ausencia de dados) a amostra foi pequeña o que pode ter influenciado, e talvez, justifique nao ter encontrado associagóes significativas com o DMO com as variáveis explicativas. De qualquer maneira, apesar dessas limitagóes, acredita-se que esse estudo apresenta potencialidades, pois revelou resultados importantes que merecem reflexao por parte da equipe assistencial que recebe o paciente ainda em fases precoces da DRC e ainda muito tem a contribuir na busca da prevengao do DMO e suas consequencias. E por último, mas nao menos importante, apesar de ter uma populagao de uma regiao específica, os resultados do nosso estudo corroboram achados de estudos internacionais já publicados.

## Conclusao

A prevalencia de DMO foi de 54,6% em pacientes com DRC em estágios nao dialíticos. Uma prevalencia acima de 50 % merece atengao dos profissionais de saúde particularmente da atengao primária, considerada pelo MS como porta de entrada no sistema de saúde nacional, no sentido de inserir o acompanhamento rigoroso desse distúrbio ainda nos estágios iniciais da DRC. Há que se considerar também que os níveis de fósforo e PTH aumentando proporcionalmente a diminuigao da TFGe é um alerta para o risco de desfechos nao só renais, como também de hiperparatireoidismo secundário a DRC e eventos cardiovasculares, por vezes mais letais para esses pacientes. O controle dessa condigao clínica, ainda se apresenta como um desafio para os profissionais de saúde que lidam com pacientes com DRC-DMO e envolve para além da assistencia, o fortalecimento de políticas públicas de saúde.
